# Supplementary material for: Infrared nanoplasmonic properties of hyperdoped embedded Si nanocrystals in the few electrons regime
Source: Nanophotonics. 2022 Jul 6;11(15):3485–93. doi: 10.1515/nanoph-2022-0283 (PMC11501647; doi:10.1515/nanoph-2022-0283)
Supplement: Supplementary file 1 — Supplementary Material Details [file j_nanoph-2022-0283_suppl.pdf]

Infrared nanoplasmonic properties of hyperdoped embedded Si nanocrystals in the few electrons regime - SUPPLEMENTARY

Meiling Zhang[1], Jean-Marie Poumirol[1], Nicolas Chery, Clément Majorel, Christian Girard, Vincent Paillard, Arnaud Arbouet, Béatrice Pécassou, and Caroline Bonafos[*]
CEMES-CNRS, Université de Toulouse, CNRS, 31055 Toulouse, France

Rémi Demoulin and Etienne Talbot
Normandie Univ, UNIROUEN, INSA Rouen, CNRS, Groupe de Physique des Matériaux, 76000 Rouen, France

Hervé Rinnert
Université de Lorraine CNRS, IJL, Nancy, France

Filadelfo Cristiano and Peter R. Wiecha
LAAS-CNRS, Université de Toulouse, CNRS, 31031 Toulouse, France

Teresa Hungria
Centre de Microcaractérisation Raimond Castaing (UAR 3623), 31400 Toulouse, France

Fabrice Gourbilleau
CIMAP, Normandie Univ, ENSICAEN, UNICAEN, CEA, CNRS, 6 Boulevard Maréchal Juin, 14050, Caen Cedex 4, France

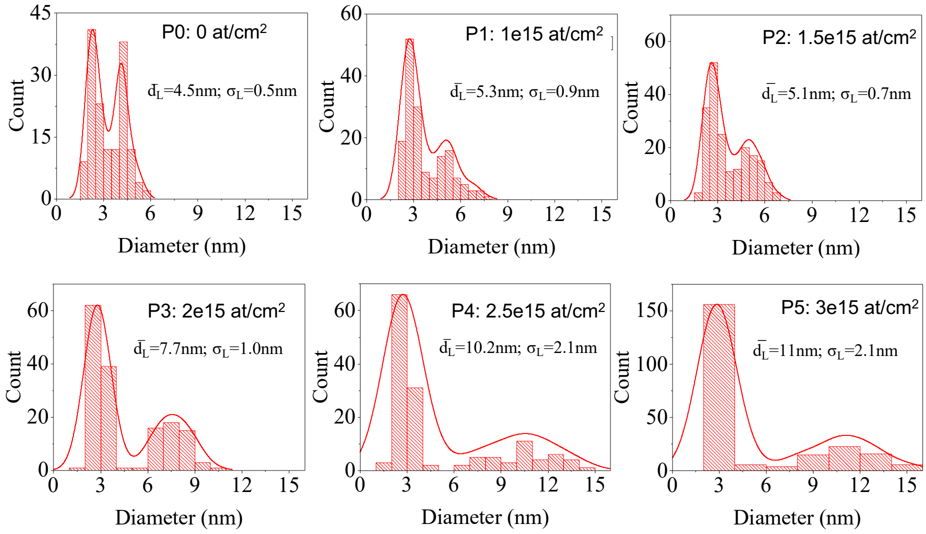

Fig. S 1: **Size distribution of NCs in the silica matrix.**
Size distribution of SiNCs in the silica matrix measured by Transmission Electron Microscopy (TEM) for all P doses. The distribution is bimodal with two characteristic diameters: a lower average value, corresponding to the undoped SiNCs located in the P profile tail, which remains constant with increasing P doses and a higher value for the doped SiNCs at the projected range increasing with increasing P doses. The average values for the doped NCs used in the simulation appear in each panel.

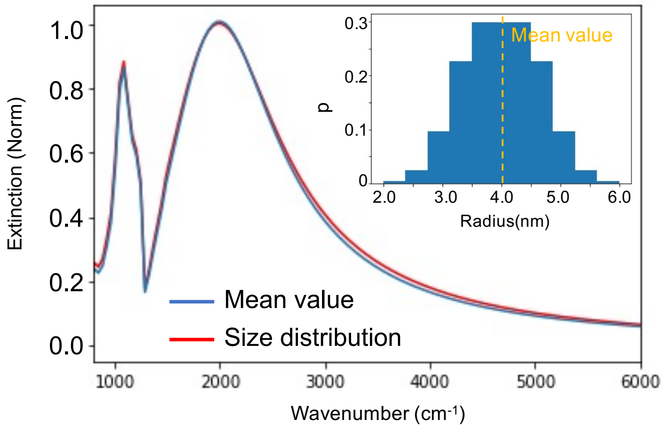

Fig. S 2: **Effect of NCs size distribution on the extinction profile**
Blue curve: calculated normalized extinction for R $= 4nm$, N $= 2 \times 10^{20} cm^{-3}$ and $\tau = 3.86 fs$. Red curve: calculated normalized extinction for a Gaussian radius distribution centered on R $= 4nm$ (as displayed in inset and made to mimic the size distribution of doped NCs in sample P3), N $= 2 \times 10^{20} cm^{-3}$ and τ calculated for each radius in the distribution using equation 4 in the main text. The two curves are nearly identical, confirming the validity our model.

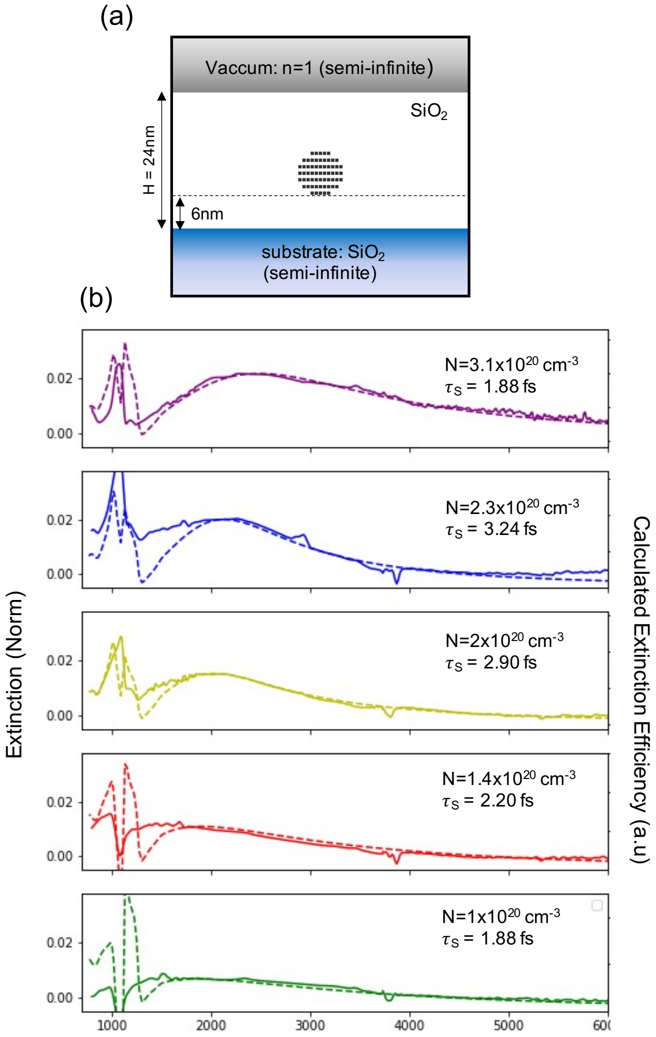

Fig. S 3: **Effect of the dielectric environnement.**
(a) Geometry used in the numerical simulations in (b), Similar to the one used in the main paper (see Figure 3) except $\epsilon_{layer}(\omega)$ is taken equal to $\epsilon_{SiO_2}(\omega)$. (b) Comparison between simulated normalized extinction (dashed line) and experimental measurement (continuous line), for all doses. The agreement between the experimental curves and the theoretical predictions is deteriorated compared to Figure 3.

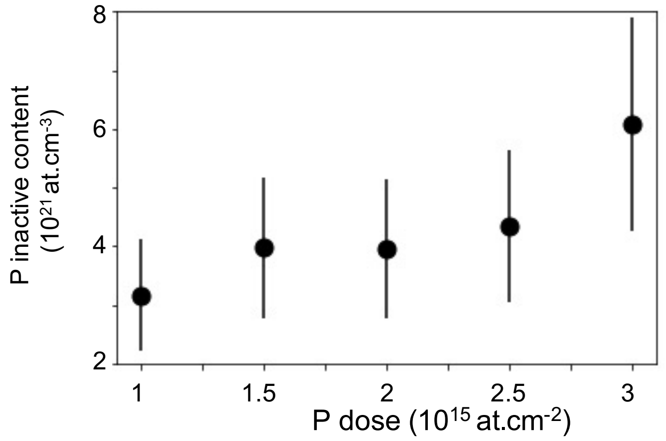

Fig. S 4: **Concentration of inactive P inside SiNCs.**
Concentration of inactive P inside big particles obtained by STEM-EDX, measured by calculating the difference between the total P concentration measured by STEM-EDX and the carrier density measured by FTIR.

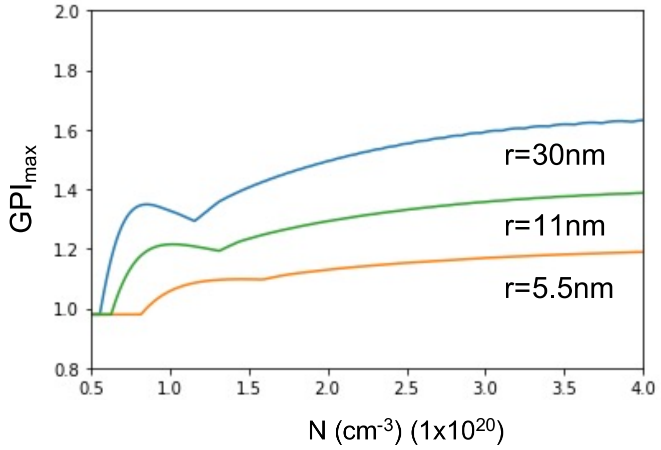

Fig. S 5: **Generalized plasmonicity index.**
Calculated maximum of the Generalized Plasmonicity Index (GPI) in the quasistatic approximation versus carrier density: $GPI(\omega) = \frac{\epsilon(\omega)-\epsilon_0(\omega)}{\epsilon(\omega)+2\epsilon_0(\omega)}$, with $\epsilon(\omega)$ the frequency dependent permittivity of the NP deduced from fitting procedure described in the main text, and $\epsilon_0(\omega)$ the dielectric function of the surrounding media deduced from equation (2). The use of quasistatic approximation is justified by the small size of the plasmonic nanostructure as compared to their resonance wavelength. We calculated the GPI_{max} for three different sizes of NC from r=5.5 nm to r= 30nm. This simulation illustrates that to improve the plasmonic behavior in our system the size of the NC is a key parameter.

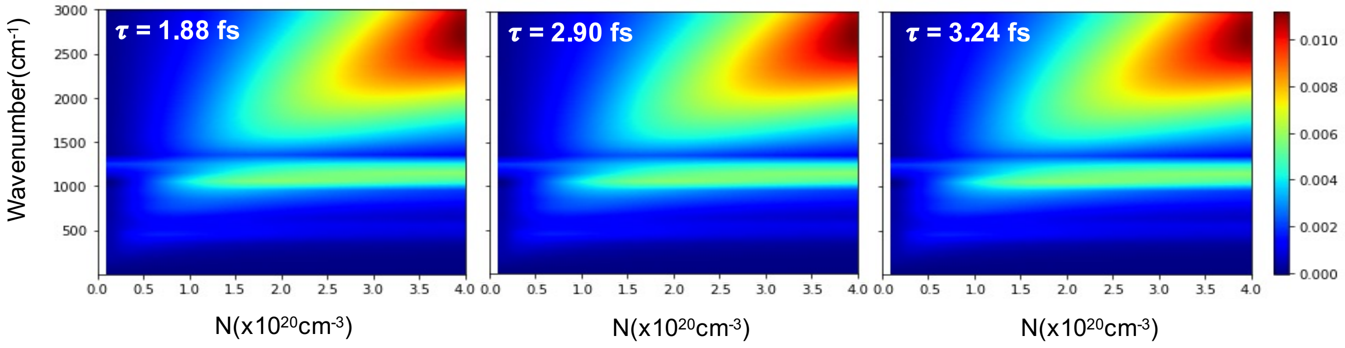

Fig. S 6: **Effect of scattering on avoided crossing.**
Color map: GDM calculations, frequency dependent extinction versus carrier density, for three different values of scattering time in the experimentally observed range.

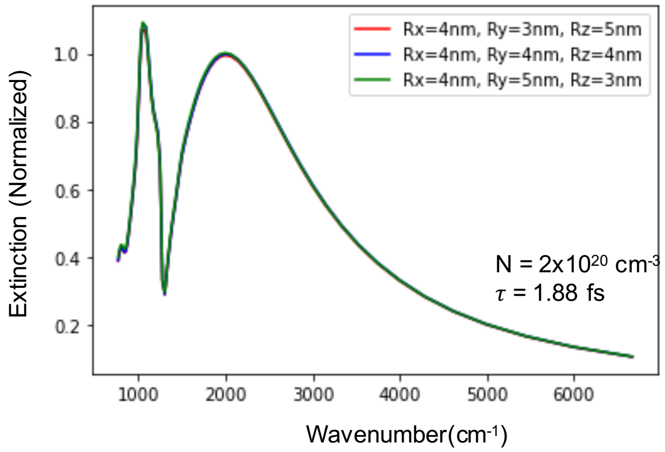

Fig. S 7: **Deviation from the spheroisity.**
Calculated normalized extinction for N $= 2 \times 10^{20} cm^{-3}$ and $\tau = 1.88 fs$, for spheroids with different geometries.
